# Recurrent Osteomyelitis Caused by Mycobacterium abscessus Necessitating Surgical Decompression and Revision Surgery With Interbody Fusion

**DOI:** 10.7759/cureus.33668

**Published:** 2023-01-11

**Authors:** Malek Bashti, Vignessh Kumar, Ian Cote, Eric C Peterson, Gregory W Basil

**Affiliations:** 1 Neurosurgery, University of Miami Miller School of Medicine, Miami, USA

**Keywords:** mycobacterium, abscessus, revision surgery, epidural abscess, atypical

## Abstract

Epidural abscesses can be caused by a number of different organisms, including atypical *Mycobacterium*. This is a rare case report of an atypical *Mycobacterium* epidural abscess requiring surgical decompression. Here, we present *Mycobacterium abscessus* causing a nonpurulent epidural collection surgically treated with laminectomy and washout and discuss clinical clues and radiologic characteristics associated with this condition.

A 51-year-old male with a past medical history of chronic intravenous (IV) drug use presented with a three-day history of falls and three-month history of progressively worsening bilateral lower extremity radiculopathy, paresthesias, and numbness. MRI demonstrated an enhancing collection at L2-3 ventral and to the left of the spinal canal causing severe compression of the thecal sac, along with heterogenous contrast enhancement of the L2-3 vertebral bodies and intervertebral disc. The patient was taken for an L2-3 laminectomy and left medial facetectomy, where a fibrous, nonpurulent mass was discovered. Cultures ultimately demonstrated *Mycobacterium abscessus* subspecies *massiliense*, and the patient was discharged on IV levofloxacin, azithromycin, and linezolid with complete symptomatic relief.

Unfortunately, despite surgical washout and antibiotic coverage, the patient presented twice more, the first time with a recurrent epidural collection requiring repeat drainage and the second time with a recurrent epidural collection with discitis and osteomyelitis with pars fractures requiring repeat epidural drainage and interbody fusion.

It is important to recognize that atypical *Mycobacterium abscessus* can cause a nonpurulent epidural collection, especially in high-risk patients such as those with a history of chronic IV drug use. Additionally, our initial intraoperative findings of a fibrous, adherent mass suggest that in cases where this entity is suspected, surgical decompression should be carefully considered. To this end, the radiologic findings associated with this condition, namely, an enhancing ventral epidural mass involving the disc space, should also be recognized. The notable postoperative course consisting of recurrent collections and osteomyelitis with a pars fracture suggests that early fusion should be considered as an option in these patients.

This case report presents clinical and radiologic findings associated with an atypical *Mycobacterium* discitis and osteomyelitis. The clinical course described herein suggests that early fusion in these patients may provide superior results to decompression alone.

## Introduction

Most often, mycobacterial infection presents with pulmonary infiltration and can be localized or disseminated. The incidence rates of nontuberculous mycobacterial (NTBM) disease have surpassed tuberculosis (TB) as the leading cause of mycobacterial lung disease in the USA. Localized infections are rare; thus, there is no standard recommended regimen for treating *Mycobacterium abscessus* osteomyelitis.

Enhancing epidural masses in the lumbar spine have a wide and varied differential diagnosis. In many cases, the differential diagnosis can be guided by imaging findings along with clinical history. For an enhancing extradural mass, the differential includes metastatic tumor, abscess, hematoma, possible vascular malformation, herniated disc fragments, and postsurgical granulation tissue [1]. The patient’s clinical presentation, time course of symptoms, and past medical history are often useful in narrowing the differential diagnosis; while hematoma and infection typically manifest over the course of hours or days, malignancy usually presents over weeks or months. Imaging is useful in further distinguishing the patient’s specific pathology. Infectious collections typically demonstrate contrast enhancement on MRI with associated enhancement of adjacent structures if a contiguous spread of the infection occurs [2]. Tumors may show contrast enhancement but are rarely associated with the enhancement of neighboring anatomy [3]. Furthermore, infectious processes tend to invade the disc space, whereas tumors rarely spare the disc space. Hematomas show contrast enhancement early after formation. While herniated discs are not contrast-enhancing, they may demonstrate some peripheral epidural contrast enhancement in the acute phase related to the inflammatory response [4]. A discitis with an adjacent epidural abscess will classically show enhancement or increased short TI inversion recovery (STIR) signal in the disc space, and abscesses typically exhibit a restricted pattern on diffusion-weighted MRI [5].

The overall incidence of infection causing localized epidural collection has been reported at an incidence of 0.2-5.1 per 10,000 cases [6,7]. The majority of epidural abscesses form posterior to the thecal sac; if epidural abscesses form anterior to the thecal sac, they typically occur in the lumbosacral spine below the level of L1 and are often secondary to discitis [8]. The most common causative organism of spinal epidural abscesses is *Staphylococcus aureus*, occurring in 63% of cases. Other common causative agents include gram-negative bacilli (16% of cases) and streptococci (9%). *Staphylococcus epidermidis* is a common cause of epidural abscess in patients with existing spinal instrumentation [9]. The specific incidence of typical and atypical *Mycobacterium* causing epidural abscess has not been reported in the literature.

To date, few cases of *Mycobacterium abscessus* causing isolated epidural abscesses have been reported, with the patients being treated with needle biopsy and intravenous antibiotics [10,11]. There is one additional reported case of *M. abscessus* causing a pacemaker pocket infection, complicated by thoracic epidural abscess requiring thoracic laminectomy and fusion [12]. Here, we present the perioperative course and multidisciplinary management of a 51-year-old male with a history of chronic intravenous (IV) drug abuse presenting with bilateral lower extremity radiculopathy and transient weakness secondary to a lumbar epidural collection caused by *Mycobacterium abscessus*.

## Case presentation

Preoperative course

**Figure 1:**
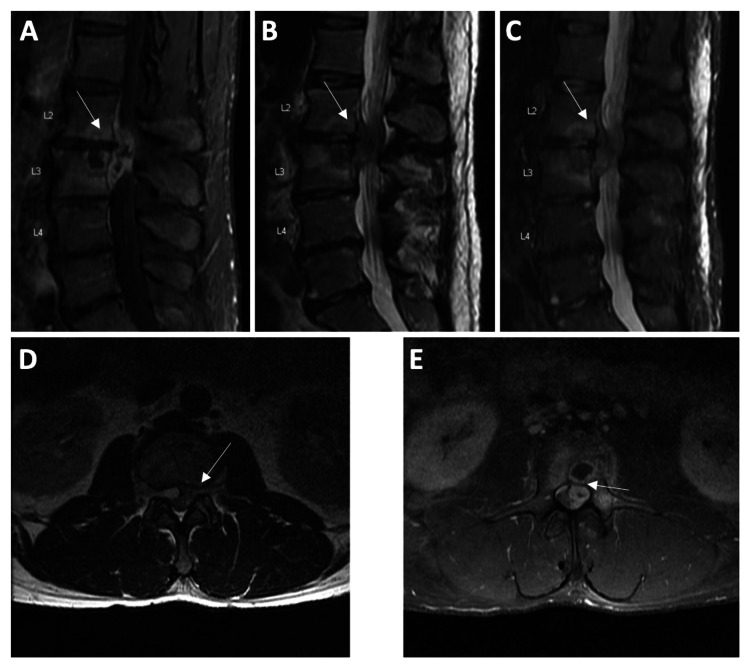
MRI with and without contrast of the lumbar spine. Sagittal T1 with contrast (A), sagittal T2 (B), sagittal T2 STIR (C), axial T1 with contrast (D), and axial T2 (E) at the level of interest. MRI shows enhancing collection at L2-3 ventral and to the left of the thecal sac, causing circumferential compression. The collection measures 38 mm (cranial-caudal) by 14 mm (anterior-posterior) by 13 mm (lateral). The inferior L2 vertebral body, L2-3 intervertebral disc, and superior L3 vertebral body demonstrate heterogenous contrast enhancement. STIR: short TI inversion recovery

A 51-year-old male presented to the emergency department with a three-day history of falls after his legs would “give out” and a three-month history of progressively worsening bilateral lower extremity radiculopathy and intermittent bilateral lower extremity numbness and paresthesias, which had become severe in nature. The patient’s informed consent was obtained for the use of non-identifiable information. He denied any perineal numbness or episodes of bowel or bladder incontinence. His past medical history was significant for chronic IV drug abuse (most recently the day prior to admission), hepatitis C, hypertension, and hypercholesterolemia. On examination, the patient was afebrile, his strength was 5/5 strength in the upper extremities and lower extremities bilaterally, his sensation was intact throughout, his patellar and Achilles reflexes were 2+ bilaterally, and there was no clonus. Post-void residual was within normal limits. MRI with and without contrast of the lumbar spine demonstrated an enhancing collection at L2-3 appearing to originate ventral and to the left of the thecal sac and causing severe canal stenosis (Figure 1). The collection measured 38 mm (cranial-caudal) by 14 mm (anterior-posterior) by 13 mm (lateral). Additionally, the inferior L2 vertebral body, L2-3 intervertebral disc, and superior L3 vertebral body also demonstrated heterogenous contrast enhancement. Laboratory studies demonstrated a white blood cell (WBC) count of 7.4 (normal: 4.0-10.5), neutrophil percentage of 69.4 (normal: 36-70), erythrocyte sedimentation rate of 8.0 (normal: 0-10), C-reactive protein (CRP) of 3.3 (normal: 0.0-0.9), and lactic acid level of 1.0 (normal: 0.0-19.8). Blood and urine cultures returned negative. Given this patient’s presentation, history of chronic IV drug use, and MRI findings demonstrating an enhancing epidural collection with the additional enhancement of the adjacent vertebral bodies and intervertebral disc, a working diagnosis of epidural abscess with discitis/osteomyelitis was made, although the differential diagnoses of herniated disc and meningioma could not be entirely excluded. As a result, the decision was made to proceed with L2-3 laminectomy with the evacuation and washout of a suspected epidural abscess (Figure 1).

Surgical technique

The patient was brought to the operating room where an open L2 and L3 laminectomy and L2-3 left-sided mesial facetectomy were performed. During the bony removal of the inferior L2 lamina, irregular fibrous tissue was immediately noted. This tissue was adherent to the ligamentum flavum but was distinct in both color and texture and appeared to be contiguous with additional fibrous tissue, which appeared to be originating ventrally. The tissue was causing the thecal sac to be displaced to the right, and care was taken to remove this tissue without causing a dural rent. The annulus of the disc space was not violated as it appeared intact and uninvolved. Once the dorsolateral fibrous tissue along with the bone and ligament was fully removed, the thecal sac was retracted from left to right, at which time a firm, beefy red mass was noted. This mass was densely adherent to the dura and spanned rostro-caudally from the bottom of the L2 pedicle to just below the L3 pedicle. Using blunt dissection, this tissue was carefully peeled away and sent for pathology. Having removed this mass, additional fibrous tissue was noted ventral to the thecal sac and was meticulously removed. Once adequate decompression was achieved, a medium Hemovac drain (Zimmer Biomet, Warsaw, IN) was placed and set to self-suction. The incision was closed in usual fashion.

Postoperative course

The patient reported complete symptomatic relief immediately postoperatively and was started on empiric cefazolin immediately postoperatively per recommendations from the infectious disease team. On postoperative day 7, cultures demonstrated the growth of acid-fast bacilli, suggesting infection with a rapid-growing *Mycobacterium* (RGM). As a result, the patient was started on empiric levofloxacin, azithromycin, and amikacin on postoperative day 7. Cultures subsequently demonstrated speciation with *Mycobacterium abscessus *subspecies *massiliense*. Susceptibilities demonstrated sensitivity to the administered antibiotic regimen. The patient was discharged on postoperative day 24 in stable condition with intravenous levofloxacin, azithromycin, and linezolid through a peripherally inserted central catheter (PICC) line. Per infectious disease recommendations, the patient was to continue antibiotics for at least three months postoperatively.

**Figure 2:**
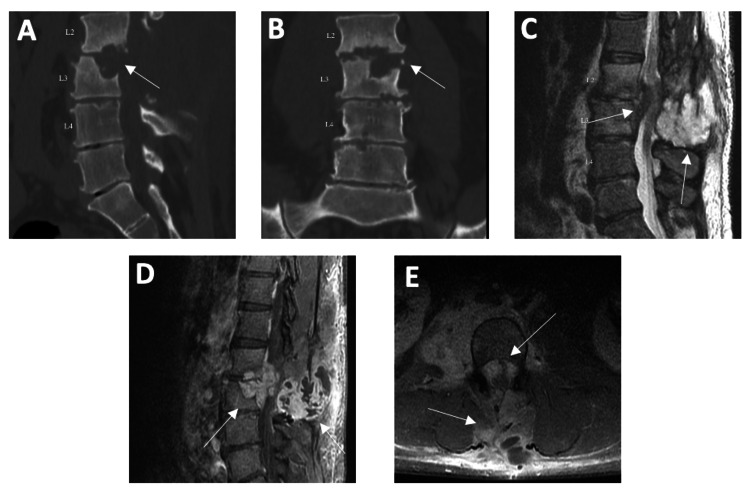
CT (A and B) and MRI (C, D, and E) of the lumbar spine taken during first rehospitalization. CT scans include sagittal T1 without contrast (top left) and coronal T1 without contrast (top middle). CT in both sagittal (A) and coronal (B) views demonstrates increased sclerosis of the endplates at the L2-3 level with endplate erosion and vertebral body destruction prominent along the inferior aspect of the L2 vertebral body consistent with a discitis and acute infection. MRI shows an epidural soft tissue lesion seen at the level of L2-3 and seen clearly on sagittal T2 (C), which also enhances on T1 post contrast (D and E) and enhances and compresses the canal and nerve roots. Also appreciated is a 5.6 × 3.8 soft tissue enhancing mass present posteriorly at this level slightly inferior to the epidural abscess, which contains multiple internal septations (E).

The patient returned to the emergency department 18 weeks later due to complains of increasing back pain and pain in the right thigh in the L2/3 distribution. On examination, he had full strength of all muscle groups of the upper and lower extremities with intact sensation. MRI demonstrated the recurrence of the epidural abscess, along with a new right psoas abscess (Figure 2). He was taken to the operating room for the evacuation of this epidural collection. After the opening of the fascia, two large pockets of purulent collection were identified and removed. The exiting L2 nerve roots and traversing L3 nerve roots were identified. With dissection to the right side, an epidural ventral phlegmon was identified and dissected off the thecal sac, which was likely causing neural compression and the pain. A component of the phlegmon was also seen tracking into the disc space and the vertebral body, and this was identified and removed. Postoperatively, he was started on amikacin, cefotoxin, and clarithromycin. Intraoperative cultures showed no growth, and he was instructed to continue this regimen for 10 weeks in total. He was seen two weeks postoperatively and reported improved pain, some continued anterior thigh numbness, and baseline strength.

**Figure 3:**
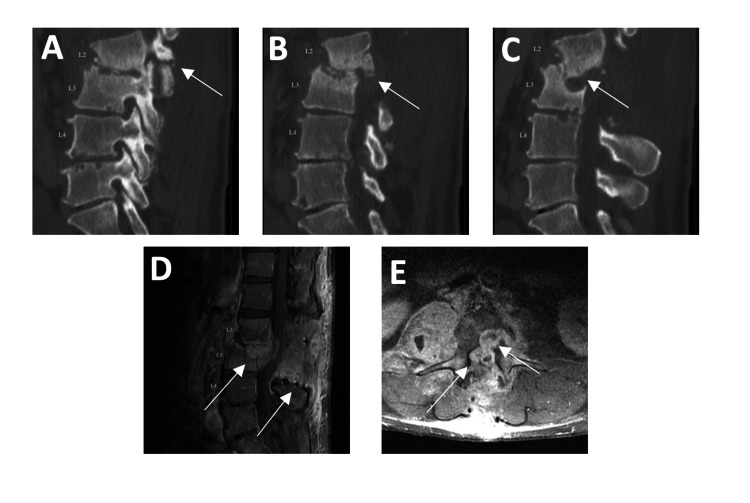
CT (A, B, and C) and MRI (D and E) of the lumbar spine taken during second rehospitalization. Significantly worsening kyphotic deformity with worsening L3 body destruction and now with retrolisthesis of L2 on L3 appreciated on both CT (A, B, and C) and MRI (D and E). MRI additionally demonstrates the extension of the abscess from the vertebral body to the canal resulting in moderate spinal canal stenosis and severe bilateral neural foraminal stenosis (D and E).

Unfortunately, the patient once again presented to the emergency department six weeks later with complaints of severe back pain with radiation into the right leg. On examination, he had full strength of all muscle groups of the upper and lower extremities with intact sensation. MRI demonstrated increasing size of the epidural abscess, increase in size of the right psoas abscess, and new fractures of the bilateral pars at L2 with anterolisthesis at L2/3. He returned to the operating room for revision laminectomy from L1 to L4 for the evacuation of the epidural abscess, L1-4 pedicle screw instrumentation, and a right retroperitoneal approach for the evacuation of the psoas collection with an L2/3 discectomy, partial corpectomies of L2/3, and the placement of a fibular strut allograft at L2/3. He was first positioned prone for the L1-4 revision laminectomy and fusion. After the decompression, pedicle screws were placed bilaterally from L1 to L4. The L1 and L4 set screws were final tightened but not at L2 and L3 to permit distraction and compression during the placement of the lateral interbody (Figure 3). Allograft was laid down in the lateral gutters. The patient was then placed in the left lateral decubitus position with the right side up. A 1.5-inch skin incision was made, and the muscle layers were dissected until the retroperitoneal fat was reached. During the dilation of the psoas, the intrapsoas abscess was encountered and drained. The disc and infected material at the L2/3 interspace were removed. The inferior 50% of the L2 body and the superior 50% of the L3 body were drilled. A cadaveric allograft strut measuring 6 cm in height, 4.5 cm in width, and 1.8 cm in anteroposterior length was made and placed in the disc space. The prior incision was then opened, and the L2/3 pedicle screws were compressed and final tightened. The infectious disease team was once again consulted, and it was recommended to continue fluconazole for one month, tigecycline for six weeks, and cefoxitin and clarithromycin for at least three months.

## Discussion

Here, we present a case of *Mycobacterium abscessus* causing a nonpurulent epidural collection in the lumbar spine in a 51-year-old male with a history of chronic IV drug use. The patient was treated with surgical drainage and IV antibiotics and had complete symptomatic relief after the third and final operation.

A literature review of *Mycobacterium* causing an epidural collection discloses a number of studies reporting both typical (*M. tuberculosis, M. bovis, *and* M. leprae*) and atypical (*M. abscessus, M. avium-intracellulare, *and* M. fortuitum*) *Mycobacterium* causing epidural abscesses. In some cases, the epidural abscess extended into neighboring bone, intervertebral disc, and paraspinal and psoas musculature [10,13,14], while in others, adjacent osseous and soft tissue structures were not reported to be involved [15-17]. As mentioned in Introduction, only one other case of an isolated *M. abscessus* epidural abscess has been reported. Given the paucity of data on *M. abscessus* causing epidural collection, the radiologic findings and risk factors are worth discussing. In this case, we observed an enhancing collection in the lumbar spine ventral to the thecal sac with the involvement of the adjacent disc space and vertebral bodies. In the other case reported in the literature [18], a similar abscess in the lumbar spine ventral to the thecal sac was seen with the enhancement of the adjacent disc space and neighboring vertebral bodies, but the involvement of the paraspinal musculature was also observed, which may represent the natural progression of infection if untreated. These radiographic findings suggest that the epidural collections caused by *M. abscessus *are typically enhancing, occur ventral to the thecal sac, involve the disc space and neighboring bony anatomy, and may progress to involve paraspinal musculature. The risk factors that predispose patients to this infection also deserve further discussion. In our case, the patient had a history of known chronic IV drug use. In the other case study, the patient denied IV drug use, although the authors report that “her behavior pattern during the hospitalization raised this possibility” [10]. This suggests IV drug use to be a major risk factor for this condition and should be a component of the history, which should raise suspicion of this diagnosis in patients with a corresponding clinical picture.

The intraoperative findings seen here are especially critical as we present the only intraoperative findings and discussion of the gross pathology of this entity. We observed an irregular, fibrous, adherent mass adherent to the ligamentum flavum and dura and contiguous with ventral fibrous tissue. Similarly, findings from the other case report, though only a biopsy was performed, revealed “reactive fibrosis … with acute and chronic inflammatory cells” [10]. These findings are especially noteworthy, as the fibrous and adherent nature of the epidural collection suggests that medical therapy alone with biopsy and IV antibiotics may be insufficient for thorough eradication. The patient treated with IV antibiotics in the other case report was lost to follow-up after discharge.

The most common mycobacterial spinal infection is *M. tuberculosis*. There are key differences between this case of *M. abscessus* infection and the characteristic *M. tuberculosis* infection, including demographic, clinical, and radiographic features. The incidence of spinal tuberculosis is highest in regions where pulmonary tuberculosis is common, primarily in Southeast Asian, Indo-Asian, and African countries [19]. *Mycobacterium abscessus *has been found to be most prevalent in East Asian countries [20]. Clinically, *M. tuberculosis *spinal involvement can be characterized by anterior wedge deformity leading to gibbus formation and vertebra plana (complete compression of the vertebral body) [19]. The clinical features of *M. abscessus* in this case were related to neural compromise: radiculopathy, paresthesias, and numbness. The radiographic characteristics of spinal *M. tuberculosis* include the involvement of the vertebral body, particularly in the paradiskal, anterior, and central components, along with sub-ligamentous involvement and sparing of the disc space [19]. Here, we find that *M. abscessus* is characterized by an enhancing collection within the spinal canal ventral to the disc space.

Epidural infectious collections may require surgical decompression and washout if neurological compromise is imminent. Surgical decision-making in these cases is critical to ensure optimal long-term outcomes. In the first surgery, we elected to forego instrumentation given our concern for active infection. In the second surgery, however, it may have been prudent to do a more aggressive discectomy and fusion rather than a second decompression alone. Indeed, the patient described in this manuscript ultimately required three surgeries due to his recurrent and resistant pathology and associated spinal instability. Based on this experience, it may have been wise to perform a full discectomy and fusion from the start to achieve adequate decompression and stabilization [20]. There is good literature on the safety and efficacy of titanium implants even in the case of active infections for spinal stabilization [21-23]. Needless to say, we do not intend to overstate the implications of this single case. Further studies to understand the general course of these rare infections are required to strengthen our findings.

Regardless of the decision to intervene, all cases of mycobacterial epidural infection require the patient to undergo a protracted treatment course with anti-mycobacterial antibiotics. Before final culture and speciation results, a broad-spectrum antibiotic regimen indicated that covering the most common bacterial etiologies (methicillin-resistant *Staphylococcus aureus*, methicillin-sensitive *Staphylococcus aureus*, gram-negative bacilli including *Escherichia coli* and *Pseudomonas aeruginosa*, and *Streptococcus* species) should be initiated. One commonly used empiric regimen is vancomycin with either cefepime or meropenem. Targeted parenteral antibiotics are typically continued for three to six months, and a repeat MRI is obtained after six to eight weeks of therapy to ensure the eradication of the infection.

Various biomarkers used in monitoring and response to the treatment of typical and atypical *Mycobacterium* can be used in assessing response to treatment of *M. abscessus*. Interleukin (IL) 1 beta, IL-6, IL-12, and tumor necrosis factor-alpha have long been used for the diagnostic identification of *Mycobacterium* and can be used in measuring effective response to treatment [24]. C-reactive protein (CRP), pentraxin-3 (PTX-3), and matrix metalloproteinase 8 (MMP-8) are other suggested markers [25]. Interferon gamma may indicate the presence of a latent infection [25]. These biomarkers were not collected in our patient; however, we believe they warrant measuring in future cases.

Spinal epidural abscess may be treated with either medical management (antibiotics) alone or surgical decompression with or without fusion in conjunction with medical management. The decision of whether to surgically decompress patients with spinal epidural abscess is multifactorial and includes the neurological function at the time of presentation and the need to obtain a pathogen to guide antibiotic treatment. The rate of failed medical management necessitating surgical decompression has been reported in the literature, typically ranging between 6% and 41%. Despite the available literature on this subject, it is not known which patients can be treated with medical management alone and which patients will require surgical decompression. However, some studies have noted that patients with diabetes, CRP > 115, WBC > 12.5, and positive blood cultures are more likely to fail medical management and require surgical intervention [7]. To this point, there is no literature assessing whether suppurative or fibrous epidural abscesses have a greater response to medical management or necessitate surgical decompression. Theoretically, a more liquid abscess without a solid component may have greater ability to dissipate after the infection is cleared, but there has been no literature to this point to date. MRI can be useful in distinguishing fibrous abscesses from suppurative abscess, as low T2 signal intensity and a capsule-like structure surrounding the periphery of the abscess can indicate that the infection has a more fibrous nature [26].

Long-term follow-up will also be needed to determine the efficacy of our surgical treatment. There is one additional case report detailing an *M. abscessus* pacemaker pocket infection complicated by subsequent thoracic epidural abscess and vertebral osteomyelitis requiring thoracic laminectomy and fusion [12]. While the operative details of that case are not provided, their decision to fuse the patient was in line with the clinical course for our patient.

## Conclusions

This case report is a rare presentation of *Mycobacterium abscessus* causing an isolated epidural collection. The patient was surgically treated with laminectomy and washout where intraoperative findings disclosed a densely fibrous, adherent mass. Postoperatively, the patient experienced complete symptomatic relief immediately and was ultimately discharged on IV levofloxacin, azithromycin, and linezolid. The fibrous nature of the epidural mass encountered in our case suggests that surgical decompression and fusion may be optimal in similar cases. Additionally, the radiologic characteristics and clinical features of this disease should be recognized.
